# *History* in the Basic Formal Ontology

**Published:** 2025-11

**Authors:** Werner Ceusters, Alan Ruttenberg

**Affiliations:** 1 University at Buffalo, 77 Goodell street, Buffalo NY 14203, USA; 2 National Center for Ontological Research, 126 Park Hall, Buffalo NY 14260, USA

**Keywords:** Basic Formal Ontology, history, change

## Abstract

The Basic Formal Ontology’s (BFO) current approach to ‘history’, in contradistinction to how the referent of ‘history’ has been described in scholarly work upon which the BFO is built, is quite reductionist: it allows only material entities to have a history, and what contributes to their histories is restricted to what takes place in the spatiotemporal region ‘occupied by’ the material parts. This has as one consequence that certain processes in which a material entity participates are not part of its history. In addition, the BFO is silent about whether instances of other types of continuants have a ‘history’. We explore how this situation came to be and propose two alternative versions for ‘history’ inclusive to all sorts of continuants currently recognized by the BFO.

## 1. Introduction

The term *History* – whenever in this paper a capitalized term is written in italics, it refers to that what is denoted by it in the Basic Formal Ontology (BFO) irrespective of in what meaning the term ‘history’ is used in the wider literature – is elucidated in BFO2020 as follows:

E1. A *History* is ‘*a Process that is the sum of the totality of Processes taking place in the Spatiotemporal Region occupied by the material part of a Material Entity [138-BFO]. Example: the life of an organism from the beginning to the end of its existence*’

[1] [capitalization ours]

A first consequence of E1 is that instances of *Site* do not have an instance of *History*. If the USA’s Advisory Council on Historic Preservation would adopt the BFO as a standard, then the BFO could be used to annotate the shooting to smithereens of an historic building as an occurrent part of the *History* of that building, but it cannot do similarly for *Processes* such as charges and withdrawals that took place on historic sites such as battlefields.

A second consequence of E1 is that processes occurring in the immaterial parts of *Material Entities* – f.i. oral cavities, anal canals and armpits – do not contribute to the *History* of that what these immaterial parts are continuant part of. This has, at first sight, some strange implications too. Kissing can under these terms not be part of one’s *History*, not even a French kiss for that matter. A patient will probably never forget an unpleasant experience such as a colonoscopy, yet, the colonoscopy itself is not part of the *History* of that patient; remembering that colonoscopy would be. Also taking a shower is not part of one’s *History*, nor the flow of the water over one’s body while showering except the absorption through the intestinal mucosa of the tiny bit of water that one accidently might have swallowed while singing.

This paper reports on the first results of an ongoing enquiry in potential solutions to this problem. It is organized as follows. Section 2 gives an overview of past and current views on what *History* denotes in scholarly work by main BFO contributors. We explain what contributed to the rather narrow view and how it is axiomatized in the First Order Logic (FOL) rendering of BFO2020. Section 3 looks at current uses of the term ‘history’ in BioPortal ontologies including the ones that imported *History* from one or another version of the BFO. The goal is to give an idea about the impact a change in the treatment of *History* in a future version of the BFO might have on ontologies that depend on it. In section 4, we elaborate on some contentious design decisions. In section 5, we start by proposing some low-hanging fruit fixes, and explore two alternative definitions for an inclusive version of ‘history’ in a future version.

## 2. The history of *History* in the BFO

The notion of ‘history’ was introduced when the foundations for the BFO were laid down, be it under the name ‘*life’*:

E2. ‘*We can define the life of a continuant as the aggregate of the occurrents in which it participates*’

[2, p44].

The scope of E2 was thus much wider than E1 thereby encompassing all *Continuants* able to participate in *Processes*, as witnessed by ‘*Qualities too have a life and participate directly in processes and indirectly in the processes in which their bearers participate*’ [2, p45].

The definition of ‘life’ along these lines was abandoned because of the (still) vague and circular elucidation of participation in BFO: ‘*p has participant c at t means: p is a process, c is a continuant and c participates in p some way at t*’ (with the further note that spatial regions do not participate in processes). *History* was introduced as a term in BFO version 2.0 [3]. It received at that time the following elucidation in the now outdated BFO 2.0 Specifications and User Guide [4], restricting the range of *Continuants* that can have a *History* to *Material Entities* and *Sites*:

E3. ‘*a history is a Process that is the sum of the totality of Processes taking place in the Spatiotemporal Region occupied by a Material Entity or Site, including Processes on the surface of the entity or within the cavities to which it serves as host [138-001]*’

[4, p70].

It was further stated that ‘*the history of a Material Entity will include, on the above account, the movements of neutrinos within the interior of the entity as they pass through*’ [4, p70]. Arp *et. al*., published around the same time, elaborates a bit further in the context of an object named ‘John’, by stating that his *History* includes also ‘*the movements of his blood cells as well as the movements of his heart and lungs and of all other constituent organs of his body*’ in addition to the, as we shall see, double-edged statement that ‘*the history of an object such as John is more than just the totality of events that might be described in John’s biography*’ [5, p123].

Noteworthy in light of elucidation E1 introduced later in BFO2020 is that the BFO2.0 specification also states that:

E4. ‘*The relation between a Material Entity and its History is one-to-one. Histories are thus very special kinds of Processes, since not only is it the case that, for any Material Entity a, there is exactly one Process which is the history of a, but also is it the case that for every History there is exactly one Material Entity which it is the history of*’

[4, p71].

E4 is noteworthy as it asserts the 1:1 correspondence between a *History* and the entity it is a history of exclusively in relation to *Material Entities* and not to *Sites*, while in Arp *et. al*. we find a slightly different account:

E5. ‘*The relation between a Material Entity and its history is one-to-one. Histories are thus very special kinds of Processes, since not only is it the case that, for any Material Entity* ***or Site****, there is exactly one Process which is its history, it is also the case that (by definition of BFO: history) there is for every History exactly one Material Entity* ***or Site*** *that it is the history of*’ [**bold** ours]

[4, p71].

E1 and E4 reflect the current situation, the latter being covered in BFO2020’s *Natural Language Specification of BFO2020* [6, p16].

### 2.1. Why such a narrow view on ‘history’?

BFO2020’s view on ‘history’ is narrow at least because of (1) the restriction that only *Material Entities* have a *History*, and (2) the exclusion of *Processes* occurring in the *Immaterial Entities* that are proper-continuant-parts of *Material Entities*. An explanation or motivation for why these restrictions have been introduced is not provided, though bits and pieces can be found online in historic documents and discussion groups. The origin of this choice is found in the working group tasked to release the OWL rendering of BFO 2.0 at the time the BFO was being revised from BFO 1.1. The intent at that time, although not included in BFO 2.0, was that there would also be ‘history segments’ - temporal parts of *History* - that allowed similar expressivity as some people wanted for temporal slices of *Continuants*, but in a way that roughly conforms to BFO [7]. The sort of things intended to be said with history segments would include things like temporary parthood: a history segment p asserted part of the history of w means that p is part of w at t, where t is the temporal region the p history segment occupies. This cannot be done without there being a 1:1 correspondence between a *History* and the entity whose history it is. However, if one did not restrict *Histories* to *Material Entities*, then the 1:1 correspondence cannot be guaranteed. The reason is that, although two *Material Entities* cannot spatially ‘overlap’ – i.e. spatially occupy the same *Spatial Region* – *Sites* can ‘overlap’ each other, and so also can *Sites* ‘overlap’ *Material Entities*, for instance the hull of a sunken ship and the portion of water that completely fills the hull. However, these considerations were made at a time the BFO was not yet fully axiomatized and an OWL formalization was in the making. The question is thus whether under the current BFO2020-FOL axiomatization, the restriction is still required. To answer this question, imagine that E1 would be replaced by the more general E1*:

E1*. A history* is a *Process* that is the sum of the totality of *Processes* taking place in the *Spatiotemporal Region* occupied by a ***Continuant***.

Note that because history* is not a term elucidated in the BFO, we don’t capitalize it.

The 1:1 correspondence in the context of E1* requires (1) each history* be the history of only one *Continuant* (1H:1C), and (2) each *Continuant* to have only one history* (1C:1H). Obviously, any *Spatiotemporal Region* in which *Processes* take place can have only one sum of these *Processes*; if such *Spatiotemporal Region* is ‘occupied by’ a *Continuant*, only then is such sum a history*. Therefore, 1C:1H is irrefutable.

One way under which 1H:1C would hold, is that the *Spatiotemporal Region str* spatiotemporally-occupied by h* is not ‘occupied by’ any other *Continuant* than *c* at all. But what if there would be a second *Continuant c2*? A problem is that ‘occupied by’, as used in E1 and copied in E1*, is a relational expression that is not defined in the BFO2020-FOL Common Logic Specification [8]. We will therefore continue to use quotes whenever referring to it and treat ‘occupied by’ as a shortcut relation which is to be interpreted as follows: *str* is ‘occupied by’ *c* if and only if (1) *str* temporally-projects-onto some *Temporal Region mtr* and spatially-projects-onto some *Spatial Region msr,* (2) at any temporal part *t* of *mtr*, *str* spatially-projects-onto some continuant part msr(t) of *Spatial Region msr*, (3) *c* only exists-at *Temporal Regions* which are temporal parts of *mtr*, and (4) at any *Temporal Region t* that *c* exists at, *c occupies-spatial-region msr(t)*. If *str* would also be ‘occupied by’ *c2*, then conditions (1) and (2) hold trivially as *c2* is not mentioned therein, and (3) holds simpliciter as many entities can exist at the same *Temporal Region*. Condition (4), however, requires further scrutiny as other axioms prevent this condition being satisfied under certain circumstances. Henceforth, references of the form [xxx-n] will correspond to axioms as such uniquely identified in BFO2020-FOL [8].

First, axiom [lzw-1] restricts the domain of occupies-spatial-region to *Independent Continuants* with the exception of *Spatial Regions*. In light of BFO2020’s current taxonomy, thus only *Material Entities (Objects, Fiat Object Parts, Object Aggregates)*, *Sites* and *Continuant Fiat Boundaries* qualify to ‘occupy’ a *Spatiotemporal Region*. Then, there are axioms that restrict certain *Continuants* to spatially occupy the same *Spatial Region* at the same *Temporal Region*. These impact satisfying condition (4) since at all times that *c* and *c2* exist, they must both spatially occupy the same *msr*. Axiom [scr-1] for instance, applied to our example, implies that if at all times *t* both *c* and *c2* instantiate *Material Entity* and spatially occupy *msr* at *t*, *c* and *c2* are continuant-part-of each other or *c* and *c2* are numerically identical. Axiom [tab-1] implies similarly that at all times *c* and *c2* instantiate *Independent Continuant* and are continuant-part-of each other, *c* and *c2* are identical, or at least one of them must be an *Object Aggregate*.

It can be shown that other combinations of what *c* and *c2* can instantiate are ruled out by axioms that specify what sorts of thing can be part of what other sort of thing (e.g. [mjj-1] and similar). What matters here is that certain combinations are still possible under the current BFO2020-FOL axiomatization, amongst which combinations involving *Sites*; *Sites* can indeed spatially occupy the same *Spatial Region* at the same *Temporal Region*. This gives us a clear answer on whether E1* is valid in the current BFO. It is not, since it allows 1C:1H to be violated.

### 2.2. Axiomatization of *History* in BFO2020-FOL

Table1 lists the axioms by means of which a substantial part of the elucidation of *History* is formally represented. Not listed is axiom [xtf-1] which states the non-identity of any universal symbol with all other universal symbols used in the axiomatization.

These axioms do not completely parallel the elucidation. Axiom [lga-1] f.i. adds to the picture that a *History* has the entity of which it is the history as participant at all the times the entity exists. What is not axiomatized is that a *History* is exclusively composed out of the processes taking place in the spatiotemporal region ‘occupied by’ the material parts of what stands in the history-of relation with the *History*. A suitable reasoner can therefore not determine algorithmically whether it is true or not that a *Process* that is asserted to be an occurrent-part of some *History h* actually is. Headache, tumor growth, teeth grinding, etc., parts of processes which occur inside a human body, certainly qualify. Walking and running, when understood as processes in which a spatial change happens as the result of the totality of bodily motions one makes to achieve the intended result, would (most likely) qualify as parts of a *History* too. Bicycling would not; only the spatial displacement and the bodily motions one makes to operate the bicycle would count, not the rolling of the wheels of the bike.

The imposed limitations are achieved by giving the relationship ‘(history-of x y)’ a central role in all *History*-related axioms except for *History’s* universal declaration [gki-1] and position in the BFO hierarchy [zuj-1]. That only *Material Entities* are to have a *History* is governed by [rph-1] and [uzz-1]. [woe-1] and [zek-1] assure the desired 1:1 correspondence.

## 3. Use of ‘history’ in publicly available ontologies

We used relevant content in the NCBO BioPortal (https://bioportal.bioontology.org/) to obtain an idea about how the term ‘history’ is used in representational artifacts submitted to it. By November 30, 2023, 124 artifacts out of the by then 1,169 submitted ones included ‘history’ as a class.

42 representational artifacts did not use the BFO as upper ontology. 18 of these 42 used the term ‘history’ without a definition; the majority of them are classifications and thesauri that do not have a coherent upper ontology at all and provide a hodgepodge of subclasses of various sorts such as historical eras and time periods (e.g. ‘pre-historic period’), sections in medical records (e.g. ‘family history’) or the sorts of assertions that are typically listed in such sections (e.g. ‘history of diabetes’), and fields of study (e.g. ‘history of feminism’). Another 18 ontologies of these 42 provide definitions that when interpreted under a BFO2020 perspective would make the term fall under *Process* (though not *History*), *Generically Dependent Continuant* or *Independent Continuant*. The remaining 6 ontologies conflate the distinction made in the BFO between *Continuant* and *Occurrent* by defining ‘history’ as ‘*the aggregate of past events; the continuum of events occurring in succession leading from the past to the present; a record or narrative description of past events*’.

82 artifacts imported *History* from the BFO, 80 of them annotated with the BFO 2.0 elucidation and two with the BFO-2020 one. Of these 82 ontologies, only 10 have at least one subclass of *History*, and only one of them a subclass which is consistent with *History*, i.e. ‘system history’ defined as ‘*the history of a system*’, ‘system’ being defined as a subclass of *Material Entity*. The inadequate use of *History* subtypes we found is shown in Table 2, together with at least one argument for why it is inappropriate.

It is safe to conclude from this analysis (1) that the word ‘history’ covers a variety of different entities in the BioPortal artifacts, (2) that BFO’s *History* is rarely used, and (3) when it is used, it is used inappropriately, with only one exception. If the BFO would change its approach to ‘history’ in a future version, impact on domain ontologies that import from the BFO would thus be minimal.

## 4. How well-founded is BFO2020’s current view on ‘history’?

The BFO is a top-level ontology intended to represent highly general categories and relations common to all scientific domains [5, p38]. Its perspective on reality, denoted by the ‘BFO Theory’ is described in the specifications [4, 6]. The Theory may not be assumed to be complete as certain general categories might have escaped the authors’ attention, are intentionally left out f.i. because some relevant science is not yet settled, or are only partly described, whether intentionally or not. Only part of the Theory is axiomatized in BFO2020-FOL. As an example, elucidation E1 is partly axiomatized by means of the axioms [zuj-1] and [rph-1], the imposed 1:1 correspondence, part of the BFO theory, is axiomatized in axioms [woe-1] and [zek-1], etc. But that it is only the material part of a *Material Entity* that determines its *History*, is not axiomatized. The axiomatization has been checked for consistency by building a model and checking whether all formulas are true in that model. But that doesn’t mean there is only one model for which all the formulas are true. If there are more, there is no guarantee that all these models are intended models. It is argued that an axiomatized ontology is ‘good’ when all satisfying models include all intended models and all intended models satisfy only what is committed to in the Theory [9]. It might thus still be the case that the axioms describe the Theory incompletely, and that the Theory matches reality incompletely. Developing the Theory, crafting the axioms, checking consistency, proving intended theorems, searching for non-intended models, all are part of a scholarly process, not just the part of the Theory concerning *History*, but the entire BFO development endeavor. Both the process itself as well as the resulting products – the Theory, definitions, axioms, etc. – can be scrutinized for their appropriateness and certain positions taken can be objected to.

### 4.1. Methodological considerations

With respect to *History*, two aspects of the process methodology may be criticized. The first one is that it is unfortunate that one has let the limitations of OWL drive the decision to impose the restrictions without providing metaphysical arguments. Indeed, in section 2.1 we explained that excluding *Sites* to have a *History* is a hack to achieve the 1:1 correspondence between a *History* and what it is the history of, and this under the view of *History* as expressed in E1. Without the 1:1 correspondence, biologists seeking to represent certain occurrent parts of the history of an organism as developmental stages would not be able to do so using OWL because of its limitations. But this is of course an argument that undermines the BFO as a realism-based reference ontology: representations should mimic reality, and not be constrained by one or other representation language. Whether *Sites* should be excluded and whether a strict 1:1 correspondence should hold, need to be decided upon by means of a metaphysical analysis.

A second objection re methodology concerns the terminology: it doesn’t seem right to use the term ‘history’ when it means ‘history of a material entity’. While it is the case that terms used as labels in the BFO are not meant to fix the meaning or assist in the interpretation of these terms outside the BFO, crafting terms that seem to denote something else than what is intended, may lead to confusion and hamper adoption as witnessed by the attempts to bring the BFO, the Ontology for General Medical Science, and Snomed CT under one framework [10]. Surely, the term ‘history’ does not have face-value if it is supposed to mean ‘history of a material entity’ as follows from E1 and axiom [rph-1].

### 4.2. Content considerations

Can the *History* component as a product be criticized? Surely, as we did in section 1. But we can also adopt a charitable interpretation of what the current *History* component is ***intended*** to be about. So, we could assume that the current BFO2020 term ‘history’ is indeed meant to be ***exclusively*** about the totality of *Processes in spatiotemporal regions occupied by a material* exactly as specified in E1 and accept that the label “History” simpliciter is misleading, something we would recommend correcting in a future version. Let us henceforth use the name ‘imep-history’, short for ‘intrinsic material entity part history’ to refer to *History* as it is now defined, and construe *history* to be broader term, assumed to be inclusive of imep, as yet to be defined.

Under this assumption, the current version of BFO would be silent (at least if we would ignore the axioms in Table 1 that restrict the relations history-of and its inverse’ range/domain) about whether other continuants, f.i. *Specifically Dependent Continuants*, *Sites*, and *Boundaries,* and even *Generically Dependent Continuants,* have histories. The 1:1 correspondence between an imep-history and what it is the history of is then (from the perspective of the BFO) appropriate.

## 5. The future of *History*?

There are a variety of considerations to be made in speculating about a possible future definition of *History*. For one thing, as our analysis has shown, there has not been much usage, perhaps because of little utility, and for sure not much correct usage. This raises the question of whether BFO should define a term that more broadly encompasses what its users understand as history, thus going back to the blackboard on how to define such a term. We could, instead, be more conservative and propose that a future version of the BFO should simply address some of what we consider oddities. A minimum would be to change the label along the lines proposed in section 4.2 and to augment the set of axioms so that they cover the intent of E1 more closely, f.i. by defining the ‘occupied by’ relation between a *Material Entity* and the *SpatioTemporal Region* spatiotemporally occupied by its history. The assumption we made in section 2.1 about the conditions that should be satisfied for ‘a history-of b’ to hold could be used as basis.

### 5.1. Substantive Improvements

Two obvious more substantive improvements come to mind. The first one is to include in the *History* of *Material Entities* certain extra *Processes* as occurrent parts, i.e. *Processes* that are not entirely taking place within the *Spatiotemporal Region str* ‘occupied by’ the material parts of the *Material Entity*, but yet in suitable ways take place in a *Spatiotemporal Region str2*. It seems that such suitability criterion – however formulated, perhaps in part by a more specific and well-defined participates-in relation – is satisfied when, for instance, John performs a colonoscopy on Jim. The movements that during a colonoscopy happen to Jim’s colon, and which under the current *History* view contribute to Jim’s *History*, are suitably related to John’s manipulations of the colonoscope which under the current view of *History* do ***not*** contribute to Jim’s *History*. Therefore, that coloscopy should be an occurrent part of Jim’s history, and also of John’s history!

A second improvement would be to expand the sorts of *Continuants* allowed to have a history, while still retaining the 1:1 correspondence. *Sites* are reasonable candidates, and for sure *Sites* that are immaterial parts of *Material Entities*. Such *Sites* can never spatially-occupy the *Spatial Region* spatially-occupied by the *Material Entity*, and thus preserve the 1:1 correspondence between the *Material Entity* and its history. Of course, such *Site s* can still spatially occupy a *Spatial Region sr* at some Temporal Region *t* which is also spatially occupied at *t* by a *Material Entity m* other than the one *s* is an immaterial part of. When *s* and *m* (1) exist throughout the same *Temporal Region mtr* and (2) spatially-occupy *sr* at all times they exist, only then would there under elucidation E1* be only one unique history* h* for which *m* and *s* both are candidates to have h* as history. But do such configurations occur in reality? They would be comparable with a scenario under which the Star Trek Enterprise’s replicator creates for Captain Picard a cup (*c*) of which the inside (s) is filled to the brim with mint tea (m) and then because of a malfunction dematerializes when he opens the replicator’s door. In such case, m, s and c exist throughout the same Temporal Region and at all times m and s exist, they spatially occupy the same *Spatial Region*. It is conceivable that in some other scenarios both m and s have h* as history, in other cases only m or only s. Future versions of the BFO could remain silent about what would be the case and leave it to domain ontologies importing history*.

Any expansion to what should be included in histories should ideally be achieved by crafting an appropriate definition and axiom set for the most general type of history under which there would be a hierarchy of specialized types, f.i. the current *History*. One possibility is to continue the line of thinking exemplified in E1*, thus keeping the *Spatiotemporal Region* ‘occupied by’ a *Continuant* as defining element, be it under a suitable interpretation of ‘occupied by’. But as the analysis in section 2.1 demonstrated, BFO2020-FOL axioms restrict the allowable sorts of Continuants so that E1* needs to reformulated as E1** to be better aligned with the axioms:

E1** A history* is a process that is the sum of the totality of (1) *Processes* P1 taking place in the *Spatiotemporal Region* ‘occupied by’ **at least one *Independent Continuant***, and (2) *Processes* P2 ‘suitably related’ to *Processes* P1.

The P2 *Processes* can then include, when properly axiomatized, *Processes* that take place in part outside the *Spatiotemporal Region* ‘occupied by’ one or more *Independent Continuants* which have a history*. E1** would then also include histories* which are the histories of certain sorts of *Specifically* (*SDC*) and *Generically Dependent Continuants* (*GDC*). Although axiom [lzw-1] limits spatial occupation to *Independent Continuants*, axiom [ild-1] allows *SDCs* and *GDCs* to be participant in *Processes*. The success of this approach will depend on the definition of suitability criteria which will probably vary considerably for the different sorts of entities, while still being consistent with the conditions that axiom [cgn-1] imposes for *SDCs* to participate in processes and [fmm-1] on *GDCs*.

### 5.2. Changing history

Another approach could be an adoption of the change theory as proposed in [11], with two different approaches to using the theory. First we give the outline of the change theory. A *change* is, in that theory, elucidated as follows:

D1. A *change* is an *Occurrent* that happens (1) to at least one *Continuant c* that is not a *Spatial Region* and (2) in a *Process p* such that in the course of *p* some particular comes in or goes out of existence or exhibits a difference in some relation to another entity, including differences in instantiation.

The proposal comes with a family of three ‘happens’ relations each one of which relates a *change* to a distinct sort of entity represented in the BFO. Roughly, changes *happen-in Processes* and *happen-to Continuants* whereby these *Continuants* participate in *Processes* (1) these *changes happen-in* or (2) occupy a *Temporal Region* that temporally overlaps the *Temporal Regions* these *changes happen-throughout*.

Two more definitions in the theory are relevant for our purposes here:

D2. *mono-sequential change*: a *change c* all whose occurrent parts that are *changes* stand in the temporal-layer-of relation to *c*

D3. *change sequence*: a *change* whose proper-temporal parts that are *mono-sequential changes* are temporally ordered

The binary relation ‘a temporal-layer-of b’ holds iff a is an occurrent part of b, both a and b exist-throughout the same temporal region, but neither instantiate temporal region. The relation is transitive and antisymmetric. A particular *p* exists-throughout *t* iff every temporal-region at which *p* exists is a temporal part of *t*.

The theory is thus far a partially axiomatized proposal which is compatible with the current axiomatization of BFO2020-FOL and its definitions and elucidations. It can thus be used as an extension. Based on these ideas, we could have a *history*+ to be elucidated as follows:

E1+. A *history*+ is a *Process* that is the sum of the totality of temporal parts of *Processes* in which a *change* happens to some instance of *Continuant*.

Elucidations D2 to D4 suggest that another candidate for an inclusive version of history focused on changes rather than processes as in the following:

E1+*. A *history*+* is a *change sequence* comprised of all *changes* that happen-to, or are suitably related to what happens-to some instance of *Continuant*.

Consider, however, that in the current axiomatization of the change theory, a changed thing is constrained to participate in a *Process* the change happens-in. Participation in a *Process* implies the change happens in a specific *Spatiotemporal Region*. But while changes have a temporal extent, not all have an obvious spatial extent, e.g. *Quality* changes. Decoupling the necessity of participation from the existence of a change opens up the possibility of more of the sort of things one would record in a biography becoming part of the history+*. Another appeal of this approach, would be that since all entities have distinct instantiations, 1C:1H would be satisfied for any kind of *Continuant*. As a motivating example, consider the Nobel committee making a decision on who will be a nominee for Economy. At the moment of that decision each nominee undergoes a change in that they acquire a role: nominee for Nobel prize. The decision and the role acquisition are suitably related, and would constitute a change sequence, which would be part of the History+*.

## 6. Conclusion

What ‘histories’ are, and what ‘histories’ can be ‘histories of’, is covered narrowly in BFO2020; changes are thus in order. While making changes to an upper ontology can have considerable consequences for reference and application ontologies that are built upon its foundations, our analysis of the current use of ‘history’ imported from the BFO in such ontologies suggests that the risk is minimal: at least for the BioPortal artefacts examined: few use the term, and if they do, then in all but one it is used erroneously. Several improvements are possible. One is purely cosmetic: changing the label ‘History’ to ‘intrinsic material entity part history’ so that it aligns better with what is actually denoted: the history of only the material parts of a *Material Entity*.

Better would be to change History to ‘history of a *Material Entity*’, which would include *Processes* in its material **and** immaterial parts as well as Processes suitably related thereto. More advanced would be to allow other continuants than Material Entities to have histories too. Therefore, we examined two alternative elucidations as candidates for an inclusive version of history. More work is required to provide a complete axiomatization of these alternatives.

## Figures and Tables

**Table 1.** Axioms in BFO-2020 about instances of *History*

ID	Comment	FOL-axiom
[gki-1]	history is a universal	(universal history)
[zuj-1]	history is subclass of process	(forall (t x) (if (instance-of x history t) (instance-of x process t))))
[lga-1]	a material entity participates in its history	(forall (h m) (if (history-of h m) (forall (t) (if (exists-at m t) (participates-in m h t)))))
[okt-1]	every material-entity has a history	(forall (m) (if (exists (t) (instance-of m material-entity t) (exists (h) (history-of h m))))
[rph-1]	history-of has domain history and range material-entity	(forall (a b) (if (history-of a b) (and (exists (t) (instance-of a history t)) (exists (t) (instance-of b material-entity t))))
[uzz-1]	material entity and its history exist at exactly the same times	(forall (m h) (if (history-of h m) (forall (t) (iff (instance-of m material-entity t) (instance-of h history t)))))
[vvy-1]	every history is the history of something	(forall (h) (if (exists (t) (instance-of h history t)) (exists (m) (history-of h m))))
[woe-1]	history-of is functional on first argument	(forall (p q r) (if (and (history-of p q) (history-of r q)) (= p r)))
[zek-1]	history-of is functional on second argument	(forall (p q r) (if (and (history-of p q) (history-of p r)) (= q r)))

**Table 2.** Inappropriate subtypes of BFO2020:*History* in BioPortal Ontologies

*History* subclasses as documented	Critique
**disease course:** *‘the totality of all processes through which a given disease instance is realized*	some of the material entities that participate in such processes surely exist prior to these processes
**experience:** *‘to conscious events in general, more specifically to perceptions, or to the practical knowledge and familiarity that is produced by these conscious processes’*	mixes processes with continuants that are the output of such processes, and such union cannot be a process
**kidney transplant status:** *‘used to specify conditions like chronic kidey* [sic] *disease’*	such a status is not a *Process,* hence not a *History*
**life course:** *‘a processual entity which has as parts all the processes in which a given organism is participant’*	participation is not time-indexed in this definition, and includes processes outside the material parts of the organism.
**life cycle:** *‘an entire span of an organism’s life, commencing with the zygote stage and ending in the death of the organism’*	assumes that an organism ceases to exist when death, otherwise it would be only part of a history and a part of history cannot be another history of the same entity
**life history:** *‘a history which includes all the processes during which resources are used by an organism to grow, survive, and reproduce over its lifetime’*	assumes (1) that such processes are the only processes that an organism *o* can participate in, and (2) that *o* ceases to exist when it stops being alive.
**provenance:** *‘the process history leading to the creation and current condition of an artifact’*	includes processes prior to the existence of the artifact and excludes processes that occur after the current condition.
**radiation attenuation:** *‘the reduction of radiation intensity upon passage of radiation through matter*	the ‘matter’ surely existed before the passage of radiation; the attenuation is thus at best only part of the matter’s history.
